# Analysis of sex difference in strychnine-intoxicated rat based on the combination of metabolic kinetics and metabolomics

**DOI:** 10.1186/s13293-025-00784-7

**Published:** 2025-11-25

**Authors:** Wen Zhang, Chaoren Wang, Haiyun Liu, Sitong Nan, Fenglin Zhang, Congying Liu, Jiangwei Yan, Juan Jia

**Affiliations:** 1 School of Forensic Medicine, Shanxi Medical University, Taiyuan, China; 2 Shanxi Province Engineering Research Center of Forensic Identification, Jinzhong, China; 3 Traffic Police Detachment of Xi’an Public Security Bureau, Xi’an, China; 4 Jinan Public Security Bureau Zhangqiu Branch, Jinan, China

**Keywords:** Strychnine, Sex, Gonadectomy, Pharmacokinetic, Metabolomics

## Abstract

**Background:**

Drug metabolism va-specific dosing. Strychnine, the primary active compound in strychnine-based alkaloids, is used for treatment of hemiplegia or amblyopia. However, knowledge of sex-based difference in the pharmacokinetics of strychnine remains limited, increasing the risk of dosage error and potential toxicity in patient.ries between men and women derived from the difference in body fat distribution and hormone secretion, necessitating sex.

**Method:**

Rats were divided into intact (possessing reproductive organ) and gonadectomized (GDX) groups, including 6 males and 6 females in each one. In the GDX rat group, testes were removed from male rat at 5 weeks of age, while ovaries were removed from female rat. The GDX rats were maintained for an additional 15 days. All intact and GDX rats were tested at 8 weeks of age. Both intact and GDX rats were subjected to acute strychnine exposure through an oral dose of 0.59 mg/kg aqueous strychnine nitrate solution. Blood sampleswere collected from orbital vein into a centrifuge tube containing sodium heparin at following time points: 5, 10, 15, 30, and 45 min, as well as 1, 1.5, 2, 4, 6, 8, 12, 24, and 48 h. In the metabolomics experiments, male and female rats were divided into experimental and control groups. Each group containing 10 males and 10 females. The experimental group was orally administered 0.59 mg/kg of aqueous strychnine nitrate, while the control group was given the same dose of ultrapure water. Blood samples were collected from the orbital vein at 30 min, 2 h, and 12 h following administration. The plasma concentration of strychnine was quantified using high performance liquid chromatography-tandem mass spectrometry (HPLC-MS/MS), while the metabolic kinetics data was acquired via HPLC-time-of-flight mass spectrometry (HPLC-TOF-MS). These data was subsequently analyzed to elucidate the intrinsic sex-specific metabolic difference between male and female rats.

**Result:**

Intact female rats metabolized strychnine more slowly than male rats, with significantly higher peak plasma concentrations. Moreover, the peak concentrations in both male and female rats decreased after gonadectomy, the plasma peak concentration in GDX female rats remained significantly higher than that in GDX male rats.The metabolic profile of the rat changed significantly after gonadectomy, suggesting that sex hormones may be involved in the metabolism of strychnine. Significant differences were also observed between the metabolomics of male and female rats, such as ABC transporter expression, pyrimidine metabolism, and linoleic acid metabolism pathways.

**Conclusion:**

Significant sex-specific difference exists between strychnine pharmacokinetics and metabolomics of male and female rats, potentially due to the differential expression of ABC transporter expression, pyrimidine metabolism and linoleic acid metabolism. These findings provide an important reference for sex-specific clinical management of strychnine toxicity.

**Plain Language summary:**

Strychnine is a medication used for the treatment of muscle weakness and visual issues. However, it affects males and females differently. If dosing is not customized according to sex, it may lead to toxicity. Therefore, we explored the mechanisms underlying the toxic effects of strychnine in male and female rats (both intact and gonadectomized). We found that female rats showed higher blood drug levels. Moreover, the peak blood drug concentrations of both male and female rats decreased after gonadectomy. Additionally, sex-specific differences were observed in the expression of ABC transporter expression, pyrimidine metabolism and linoleic acid metabolism. These differences may explain why strychnine affects male and female rats differently.

**Supplementary Information:**

The online version contains supplementary material available at 10.1186/s13293-025-00784-7.

## Highlights

The plasma concentration of strychnine is significantly lower in GDX rat than that in intact rat.

The primary metabolites showing differences between male and female rats after strychnine exposure.

Different expression of ABC transporters, pyrimidine metabolism and linoleic acid metabolism between sexes may be responsible for the difference of strychnine absorption and metabolism.

**Supplementary Information:**

The online version contains supplementary material available at 10.1186/s13293-025-00784-7.

## Background

Strychnine, the primary active compound in strychnine-based alkaloids, selectively stimulate the central nervous system, increasing skeletal muscle tension. It is commonly used in the treatment of optic atrophy, facial nerve paralysis, and certain cancers and tumors [1–4]. However, excessive dosage lead to tonic convulsions and even respiratory paralysis, which can be life-threatening [5], making the clinical use of strychnine extremely limited.

Sex-based difference is an important factor in the study of drug metabolism. Men and women show significant differences in biology, physiology, and hormone profile [6–9]. A clinical pharmacology report indicates that 31% of studies show a potential influence of sex on drug metabolism, based on pharmacokinetic (PK) differences of >20% between males and females [10]. In the same report, PK measurements for 11 drugs showed >40% differences. In pharmacology, these differences affect drug absorption, distribution, and metabolism, potentially leading to difference in metabolic profiles [11]. In toxicology, they affect toxicity assessments and cause deviations in dose determination. Garratt studied the effects of sex differences on 17-α estradiol (17aE2) by castrating male mice and ovariectomizing female mice prior to 17aE2 treatment. The results demonstrated that nearly all sex-specific metabolic responses to 17αE2 were either inhibited or attenuated in male mice [12]. Jaclyn conducted gonadectomy (castration in males and ovariectomy in females) to evaluate the influence of gonadal hormones on ketamine metabolism in mice. The study revealed that ovariectomy did not significantly alter ketamine metabolism in female mice, whereas orchiectomy modified the pharmacokinetic profile in male mice [13]. These findings suggest that gonadectomy significantly impacts sex difference studies in metabolic and pharmacological research.

Current research on strychnine mainly focuses on its detection in protozoa, metabolite profiles [14], adverse clinical effects, and toxicokinetics [15]. Few studies have explored sex-based difference in strychnine metabolism kinetics and metabolomics. To address this gap in the literature, we analyzed sex-specific difference of strychnine metabolism in male and female rats. By comparing PKs and metabolomics profiles before and after gonadectomy, we aimed to identify the factors underlying these differences.

## Methods

### Chemicals and reagents

Strychnine nitrate (Suzhou Yake Chemical Reagents Co. Ltd.), acetonitrile (Sigma, USA), methanol (Sigma, USA), and ammonium acetate (Sigma, USA) were used. All chemicals and solvents were of American Chemical Society or high-performance liquid chromatography (HPLC) grade.

### Animal model and treatment

#### PKs animal model Preparation

In detail, 6 intact 8-week-old male and female Sprague-Dawley (SD) rats, as well as 6 intact 5-week-old male and female SD rats were purchased from Changyang Xishan Breeding Farm (Beijing Changyang Xishan Breeding Farm, China). All rats were provided standard chow and freely-available drinking water. They were acclimatized for 1 week and fasted overnight before drug administration. The rats were divided into intact and GDX groups, with 6 males and 6 females in each group. In GDX group, testes were surgically removed from male rat at 5 weeks of age, while ovaries were excised from female rat. Following surgery, GDX rats were maintained for an additional 15 days before testing. All animals, both intact and GDX, were tested at 8 weeks of age.

Both intact and GDX rats were subjected to acute strychnine exposure through an oral dose of 0.59 mg/kg aqueous solution of strychnine nitrate. Blood was collected from orbital vein into a centrifuge tube containing sodium heparin at following time points: 5, 10, 15, 30, and 45 min, as well as 1, 1.5, 2, 4, 6, 8, 12, 24, and 48 h. The collected blood was shaken well and centrifuged at 1500 g for 10 min to separate plasma, then stored at −20 ℃ until use. All experimental procedures were reviewed and approved by the Ethical Committee for Use of Laboratory Animals of the Shanxi Medical University (SYDL2025013).

#### Metabolomics animal model Preparation

Male and female SD rats were divided into experimental and control groups. Each group contained 10 male and 10 female rats. The experimental group was orally administered 0.59 mg/kg of aqueous strychnine nitrate, while the control group was given the same dosage of ultrapure water. Blood was collected from orbital vein into sodium heparin-coated centrifugal tubes pre-chilled at 4 ℃, at 30 min, 2 h, and 12 h post-administration respectively. Plasma was separated by gentle inversion, immediately quenched in liquid nitrogen, and centrifuged at 1500 g for 10 min at 4 ℃. The prepared plasma was stored at − 80 ℃ until LC-MS/MS analysis.

### Sample Preparation

#### PKs sample Preparation

In detail, 180 µL of plasma was pipetted into a tube, followed by the addition of 20 µL of internal standard (100 ng/mL ephedrine) and the mixture was vortexed for 5 s. Next, 1 mL of acetonitrile precipitation protein was added to this mixture, which was vortexed again for 20 s. Finally, the mixture was centrifuged at 13,000 g for 10 min and the supernatant was transferred to a clean tube. This supernatant was evaporated to dryness under a stream of nitrogen at 35 ℃, then reconstituted in 200 µL of a compound solution consisting of methanol and 10 mM/mL ammonium formate (containing 0.1% formic acid) in a 40:60 (v/v) ratio. The reconstituted supernatant was stored at −80 ℃ until use. Subsequently, the supernatant was passed through a 0.22 μm organic filter before analysis using 1260–6470 HPLC-tandem mass spectrometry (MS/MS).

The matrix effect was evaluated by comparing the peak areas of strychnine spiked into post-extracted blank matrices with those of neat standard solutions. Following processing according to the established method, the residues from 5 blank plasma and 5 blank liver samples were dried under nitrogen and prepared for further analysis. The nitrogen-dried residue from each sample was reconstituted in 3 equal portions and spiked with standard solutions to achieve low (0.5 ng/mL), medium (10 ng/mL), and high mass (300 ng/mL) concentrations. After injection and analysis, peak area A was obtained. The standard mixed solutions of low, medium, and high mass concentrations were prepared. After injection and analysis, peak area B was obtained. The matrix effect was calculated as the ratio of A to B.

#### Metabolomics sample Preparation

The stored plasma samples were thawed at 4 °C, and 100 µL of each sample was transferred to a centrifuge tube. Proteins were precipitated using 1 mL of methanol/acetonitrile/water solution (2:2:1, v: v:v) at 4 °C. After vortex mixing, the sample mixture was sonicated for 30 min (ice was used to maintain the low-temperature conditions). The sonicated mixture was allowed to stand for 10 min at 20 °C, then centrifuged at 14,000 g for 20 min at 4 °C. The resulting supernatant was collected and dried under vacuum. For instrumental analysis, the dried supernatant was reconstituted in 100 µL of aqueous acetonitrile solution, vortexed, then centrifuged at 14,000 g for 15 min at 4 °C. To ensure instrument stability and data reliability, quality control samples were prepared and analyzed using random sampling.

### Instrumental analysis

#### HPLC-MS/MS analysis

Chromatographic conditions: separation was performed on a ZORBAX Eclipse XDB-C18 (2.1 × 150 mm, 3.5 μm) column at 20 ℃, with a flow rate of 0.2 mL/min and an injection volume of 5 µL. The mobile phase consisted of A: water + 10 mM/mL ammonium formate + 0.1% formic acid, and B: methanol. The gradient elution program was as follows: 0–1 min, 20% B; 1–6.5 min, 20%−80% B; 6.5–7.5 min, 80% B; 7.5–8.5 min, 80%−20% B; 8.5–12 min, 20% B.

MS conditions: Detection was carried out using an Agilent Jet Stream electrospray ionization source in positive ion mode, operating in multiple reaction monitoring mode. The ion source temperature was 300 ℃, and the nebulizer temperature was 305 ℃. The gas flow rate was maintained at 11.0 L/min, with the capillary voltage of 4000 V.

#### Metabolomics analysis

Chromatographic conditions: separation was performed in a Waters Acquity UPLC BEH Amide column (1.7 μm, 2.1 × 100 mm). The column temperature was 25 ℃ and flow rate was 0.5 mL/min. The injection volume was 2 µL, with the mobile phases consisting of A: water + 25 mM ammonium acetate + 25 mM ammonia, and B: acetonitrile. The gradient elution program was as follows: 0–0.5 min, 95% B; 0.5–7 min, 95%−65% B; 7–8 min, 65%−40% B; 8–9 min, 40% B; 9–9.1 min, 40%−95%B; 9.1–12 min, 95%B.

MS conditions: Detection was carried out using electrospray ionization in both positive and negative ion modes. The following parameters were used: spray voltage, ± 5500 V; ionization temperature, 600 ℃; nebulization air pressure, 60 psi; auxiliary air pressure, 60 psi; air curtain air pressure, 30 psi; time-of-flight MS scanning range (*m/z*), 60–1000 Da; product ion scanning range, 25–1000 Da. The secondary mass spectra were acquired using information-dependent acquisition in high-resolution mode. The cone bore voltage was ± 60 V (positive and negative modes) and collision energy was 35 ± 15 eV.

### Data analysis

Statistical analyses were performed using GraphPad Prism software (Version 8.0.2; GraphPad Software, Inc., USA). Paired-sample t-test was employed to assess intergroup differences. A significance threshold of *p* < 0.05 was established, with values below this threshold considered statistically significant. All data was presented as mean ± standard deviation (SD). To ensure methodological reliability, all experiments were independently replicated 3 times.

## Results

### Method validation

Method validation for strychnine detection in rat whole blood was performed prior to experimental procedures. Analytical results demonstrated that the whole blood matrix from control rat did not cause interference with strychnine detection. The method exhibited good linearity within the concentration range of 0.25–300 ng/mL. The assay demonstrated acceptable performance in terms of intra-day and inter-day accuracy and precision, extraction recovery, and matrix effects at 3 quality control concentrations (0.5, 10, and 300 ng/mL) as summarized in Table 1.

**Table 1:** Intra-day and inter-day precision, Accuracy, extraction recovery, and matrix effect of STR(Data are Mean ± SD, *n* = 6)

Compound	LOD (ng/mL)	Conc(ng/mL)	Accuracy (%)	Inter-day precision (%)	Intra-day precision (%)	Extraction recovery (%)	Matrix effect (%)
STR	0.1	0.5	101.91 ± 1.93	2.45	4.62	93.83 ± 10.13	87.72 ± 3.02
		10	95.50 ± 1.81	2.75	3.34	88.11 ± 1.87	93.26 ± 1.53
		300	93.17 ± 2.16	2.57	3.87	88.99 ± 1.41	94.75 ± 1.78

### Metabolic kinetic analysis in rat

**Fig. 1:**
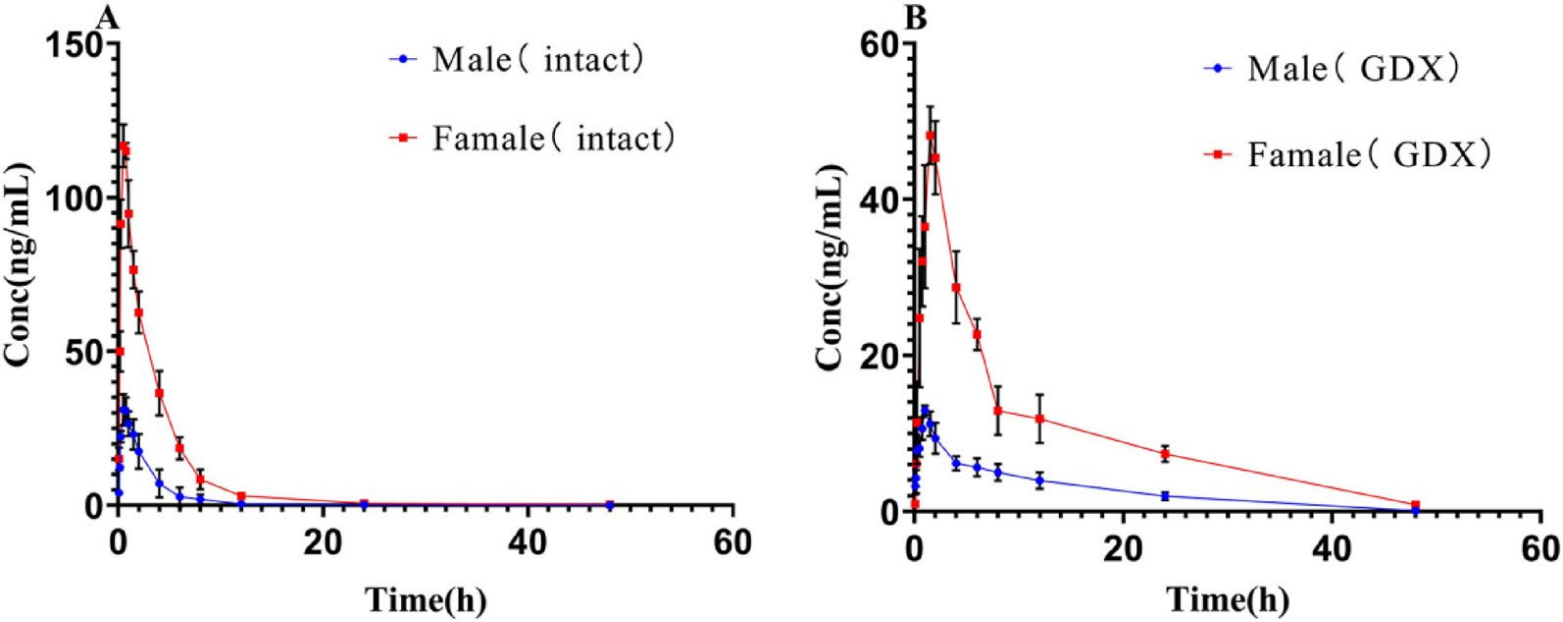
Concentration-time profiles of strychnine (oral administration, 0.59 mg/kg) in male and female rats in the intact group and GDX group (*n* = 6, each). **A**: intact group and **B**: GDX group

The PK model was established using DAS 2.0 PK software, with time as the abscissa and average plasma concentration of strychnine as the ordinate. Variance analysis of plasma strychnine concentrations in intact and GDX male and female rat was conducted using IBM SPSS Statistics 26 software, and significant difference was observed between the sexes (Fig. 1; Tables 2 and 3).

**Table 2:** Pharmacokinetic parameters of strychnine on intact male and female rats(Data are Mean ± SD, *n* = 6)

Parameter	Unit	Male	Female
Tmax	h	0.58 ± 0.13	0.63 ± 0.14
Cmax	ng/mL	33.37 ± 2.01	116.44 ± 2.70****
AUC(0-t)	ng*h/mL	96.77 ± 24.87	408.76 ± 58.40****
AUC(0-∞)	ng*h/mL	96.96 ± 24.73	403.39 ± 57.73****
t1/2	h	3.84 ± 1.48	5.70 ± 2.60

The data was given as mean ± SD(*n* = 6); ****p<0.0001

**Table 3:** Pharmacokinetic parameters of strychnine on GDX male and female rats(Data are Mean ± SD, *n* = 6)

Parameter	Unit	Male	Female
Tmax	h	1	1.60 ± 0.22*
Cmax	ng/mL	13.00 ± 0.60	48.07 ± 4.06****
AUC(0-t)	ng*h/mL	151.79 ± 10.38	489.27 ± 40.60****
AUC(0-∞)	ng*h/mL	152.91 ± 10.34	501.07 ± 43.47****
t1/2	h	7.68 ± 0.94	9.06 ± 1.82

The data was given as mean ± SD(*n* = 6); *p<0.05and****p<0.0001

The optimal atrioventricular model was selected based on the Akaike Information Criterion (AIC) and the coefficient of determination (R2). The smaller the AIC value and the closer R2 is to 1, the better the atrioventricular model. Pharmacokinetic analysis demonstrated that intact male rats were best described by a two-compartment model, whereas both intact and GDX female rats followed three-compartment kinetics; GDX male rats exhibited one-compartment kinetics. In intact group, the peak of plasma concentration in female rats was 3.49 times in male rats. Female rats reached this concentration 0.05 h later than male rats. In GDX group, the peak of plasma concentration in female rats was 3.70 times in male rats. Female rats reached this concentration 0.6 h later than male rats. The area under the curve (AUC) of female rats was significantly higher than that of male rats before and after gonadectomy.

### Analysis of rat metabolomics models

**Fig. 2:**
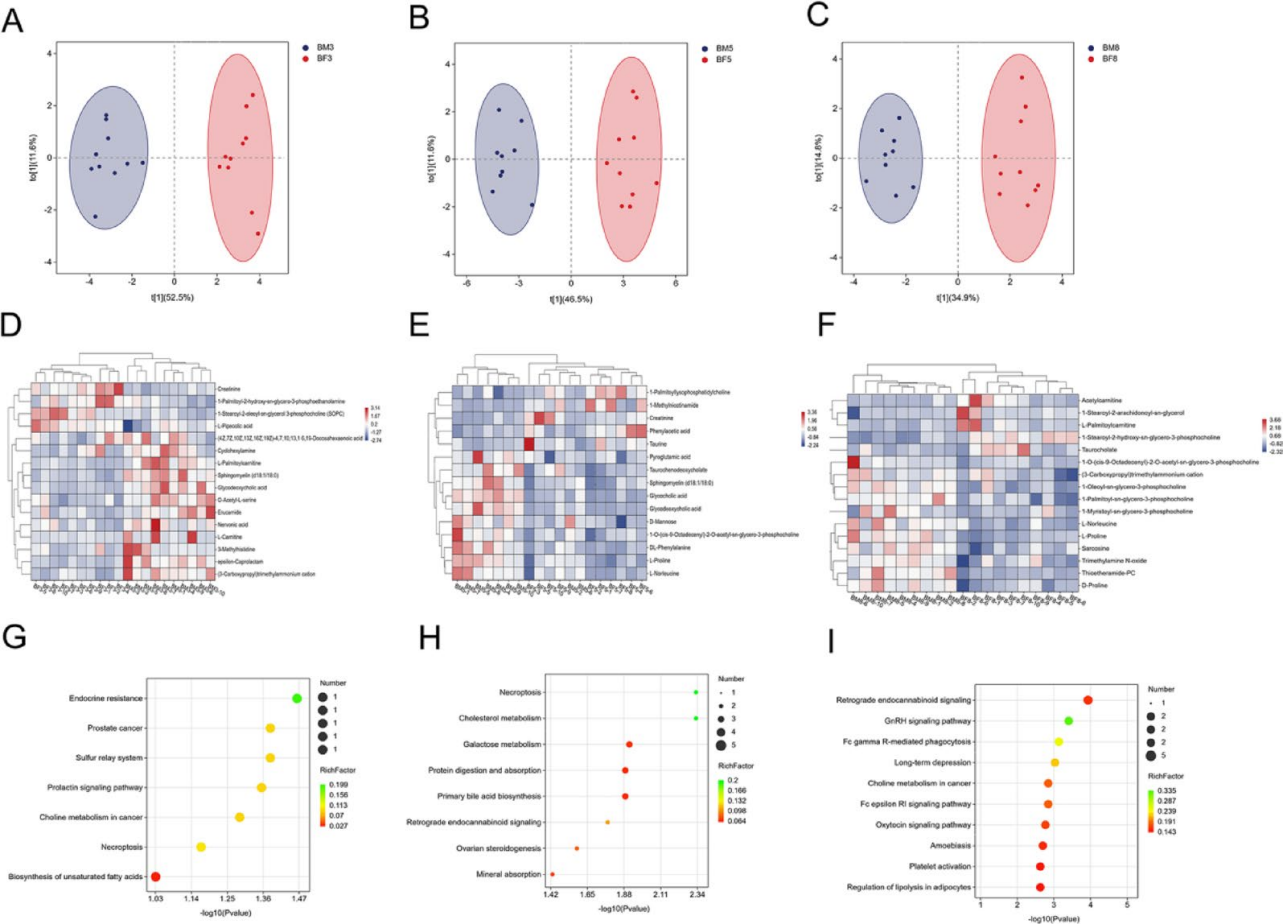
Metabolomics of male and female rats in the control group at 30 min, 2 h, and 12 h OPLS-DA analysis of differential metabolites revealed gender-dependent separation in the metabolomics of control-group rats (blue indicates males, red indicates females). **A**: 30 min (BM3: Metabolic results of control male rats with 30 min; BF3: Metabolic results of control female rats with 30 min), **B**: 2 h (BM5: Metabolic results of control male rats with 2 h, BF5: Metabolic results of control female rats with 2 h), C: 12 h (BM8: Metabolic results of control male rats with 12 h, BF8: Metabolic results of control female rats with 12 h). Heatmap of differentially metabolized compounds in male and female control rat. Blue indicates reduced expression levels, white indicates no regulation, and red indicates increased expression levels. **D**: 30 min, **E**: 2 h, **F**: 12 h. Perform metabolic pathway analysis on the filtered differentially expressed metabolites using the KEGG database. **G**: 30 min, **H**: 2 h, **I**: 12 h

Orthogonal partial least squares-discriminant analysis (OPLS-DA) were computed for control female and male rats. The OPLS-DA results demonstrated significant separation among the 3 selected time points in both sexes (Fig. 2A-C). Differential metabolites were identified using stringent criteria: variable importance in projection (VIP) scores > 1 and statistically significant from t-test. These results were derived from the OPLS-DA model. Metabolite profiling identified 16 compounds at 30 min, 15 at 2 h, and 16 at 12 h post-administration (specific compounds listed in Appendix 1). The top 20 most significant metabolites were selected for further analysis, and gender-specific heatmaps were generated (Fig. 2D-F). Kyoto Encyclopedia of Genes and Genomes(KEGG)pathway enrichment analysis of these selected metabolites revealed substantial differences in plasma metabolic profiles between control male and female rats (Fig. 2G-I).

**Fig. 3:**
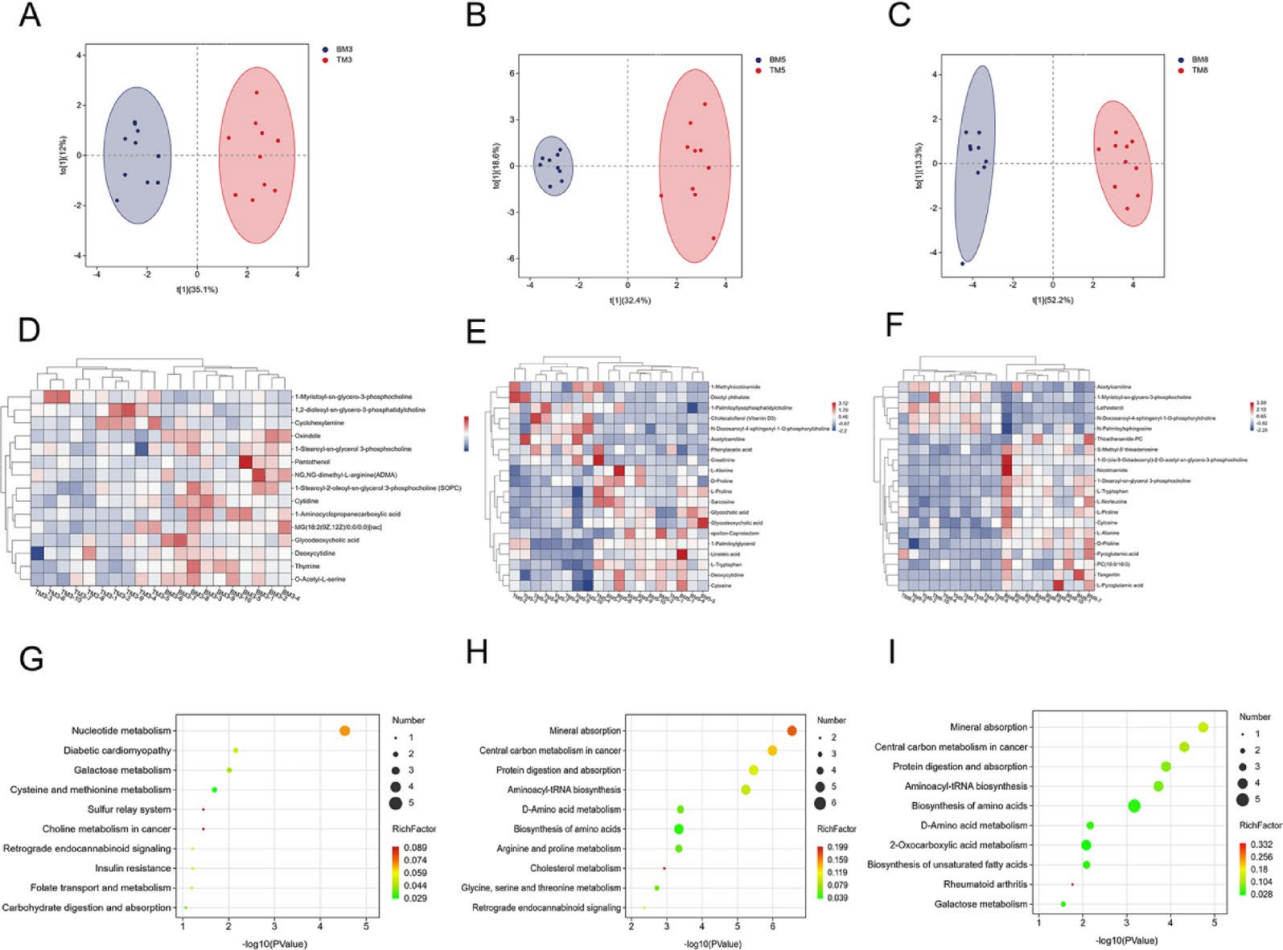
Metabolomics of male rats in the control and experimental group at 30 min, 2 h, and 12 h Analysis of differential metabolites via OPLS-DA revealed differences in metabolomics data between male rats in the control and experimental groups (blue indicates control group, red indicates experimental group). **A**: 30 min (TM3: Metabolic results of experimental male rats with 30 min), **B**: 2 h (TM5: Metabolic results of experimental male rats with 2 h), **C**: 12 h (TM8: Metabolic results of experimental male rats with 12 h). Heatmap of differentially metabolized compounds in male rats in the control and experimental groups, Blue indicates reduced expression levels, white indicates no regulation, and red indicates increased expression levels. **D**: 30 min, **E**: 2 h, **F**: 12 h. Perform metabolic pathway analysis on the filtered differentially expressed metabolites using the KEGG database. **G**: 30 min, **H**: 2 h, **I**: 12 h

OPLS-DA scores were calculated for the control group and experimental male rats. The OPLS-DA analysis revealed significant metabolic separation across the 3 examined time points (30 min, 2 h, and 12 h) between control and experimental groups (Fig. 3A-C). Differential metabolites were identified using stringent selection criteria: VIP scores > 1.0 and *P* < 0.05, as derived from the OPLS-DA model.

Metabolite profiling identified 15, 29, and 29 compounds at 30 min, 2 h, and 12 h post-treatment, respectively. The 20 metabolites showing the most significant changes were selected for further analysis. (complete list provided in Appendix 2), and their expression patterns were visualized in a heatmap (Fig. 3D-F). Subsequent KEGG pathway enrichment analysis demonstrated substantial alterations in plasma metabolic pathways between control and experimental male rats (Fig. 3G-I).

**Fig. 4:**
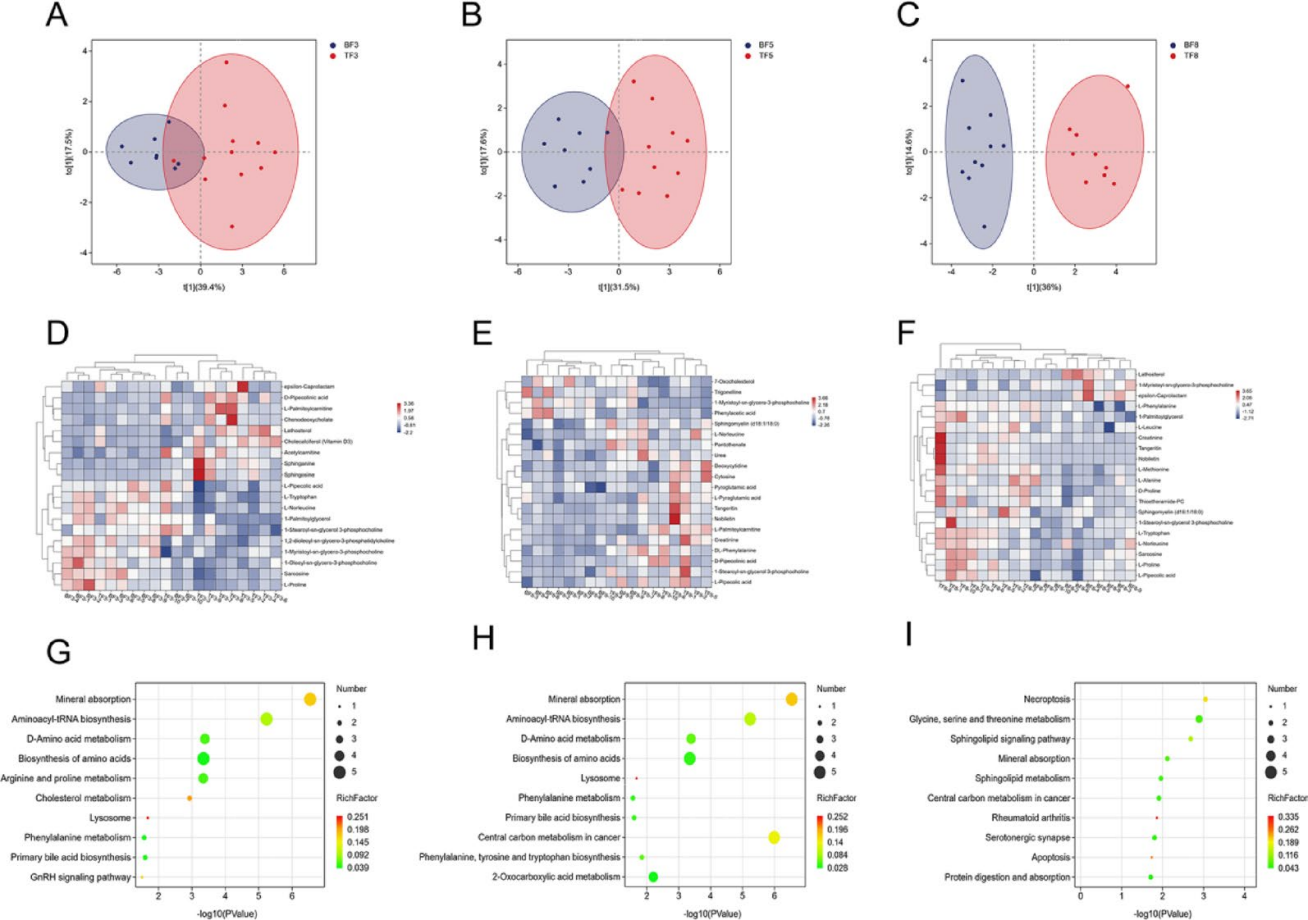
Metabolomics of female rats in the control and experimental group at 30 min, 2 h, and 12 h Analysis of differential metabolites via OPLS-DA revealed differences in metabolomics data between female rats in the control and experimental groups (blue indicates control group, red indicates experimental group). **A**: 30 min (TF3: Metabolic results of experimental female rats with 30 min), **B**: 2 h (TF5: Metabolic results of experimental female rats with 2 h), **C**: 12 h (TF8: Metabolic results of experimental female rats with 12 h). Heatmap of differentially metabolized compounds in female rats in the control and experimental groups, Blue indicates reduced expression levels, white indicates no regulation, and red indicates increased expression levels. **D**: 30 min, **E**: 2 h, **F**: 12 h. Perform metabolic pathway analysis on the filtered differentially expressed metabolites using the KEGG database. **G**: 30 min, **H**: 2 h, **I**: 12 h

OPLS-DA was performed to compare control and experimental female rats. Significant metabolic differences were observed between the groups at the three time points examined (30 min, 2 h, and 12 h). Differential metabolites were identified based on the OPLS-DA model using VIP scores > 1.0 and p-values < 0.05 as selection criteria. (Fig. 4A-C). Metabolite profiling identified 19, 25, and 26 compounds at 30 min, 2 h, and 12 h post-treatment, respectively. The top 20 most significantly altered metabolites (complete list is provided in Appendix 3) were selected for subsequent analysis, and their expression patterns were visualized using a heatmap (Fig. 4D-F). Significant changes in plasma metabolic pathways between control and experimental female rats were further revealed by KEGG pathway enrichment analysis (Fig. 4G–I).

**Fig. 5:**
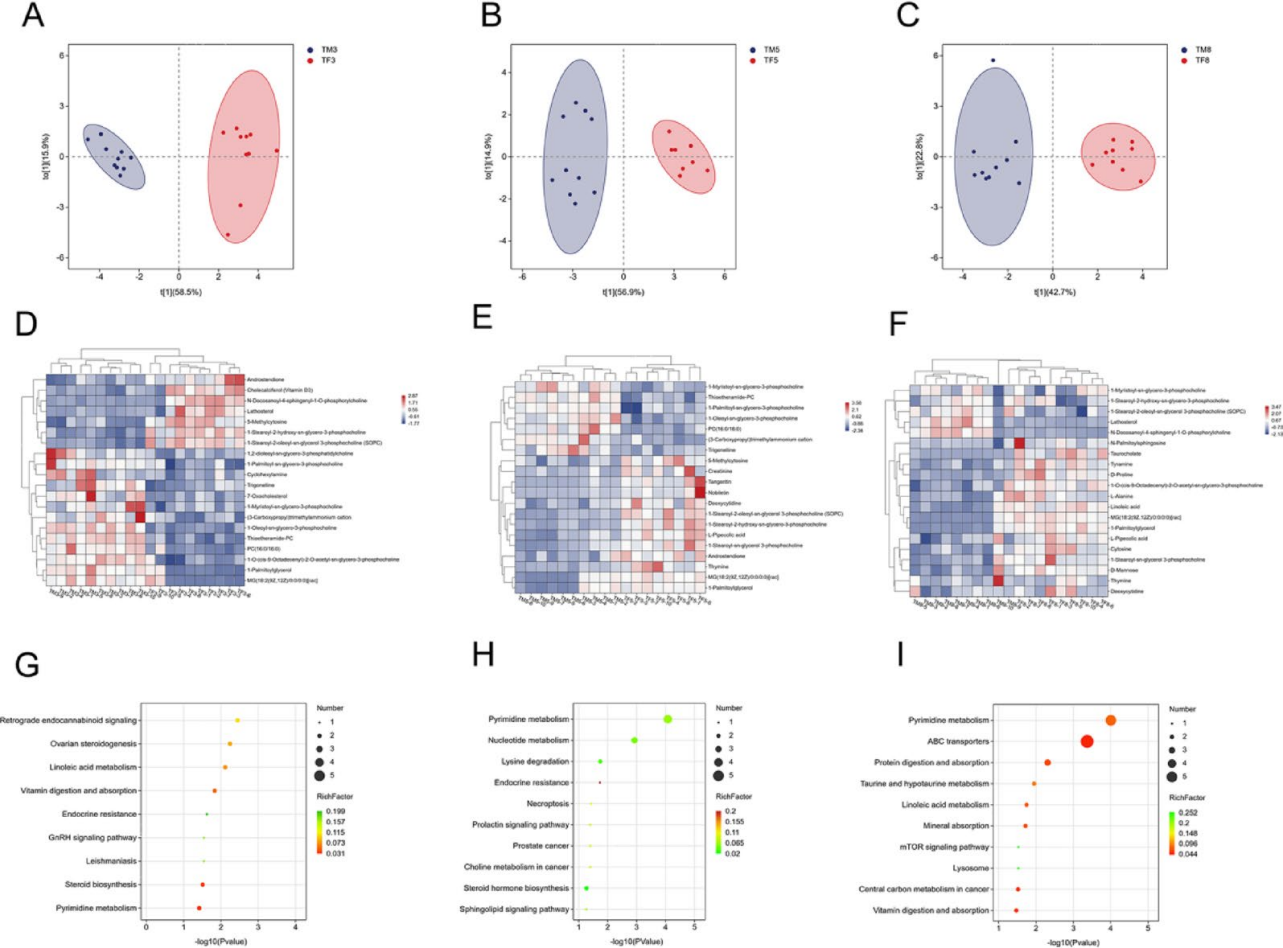
Metabolomics of male and female rats in the experimental group at 30 min, 2 h, and 12 h OPLS-DA analysis of differential metabolites revealed gender-dependent separation in the metabolomics of experimental rats (blue indicates males, red indicates females). **A**: 30 min, **B**: 2 h, **C**: 12 h. Heatmap of differentially metabolized compounds in male and female experimental rats, Blue indicates reduced expression levels, white indicates no regulation, and red indicates increased expression levels. **D**: 30 min, **E**: 2 h, **F**: 12 h. Perform metabolic pathway analysis on the filtered differentially expressed metabolites using the KEGG database. **G**: 30 min, **H**: 2 h, **I**: 12 h

OPLS-DA scores were calculated for female and male experimental rats across three time points, revealing significant metabolic separation in the model (Fig. 5A–C). Using strict criteria (VIP > 1.0, *P* < 0.05), differentiated metabolites were identified, with 24, 25, and 35 compounds detected at 30 min, 2 h, and 12 h, respectively. The top 20 metabolites (complete list is provided in Appendix 4) were selected for further analysis. A gender-specific metabolite heatmap was generated (Fig. 5D–F), and KEGG pathway enrichment analysis of the selected metabolites revealed significant differences in plasma metabolites between sexes (Fig. 5G–I).

**Fig. 6:**
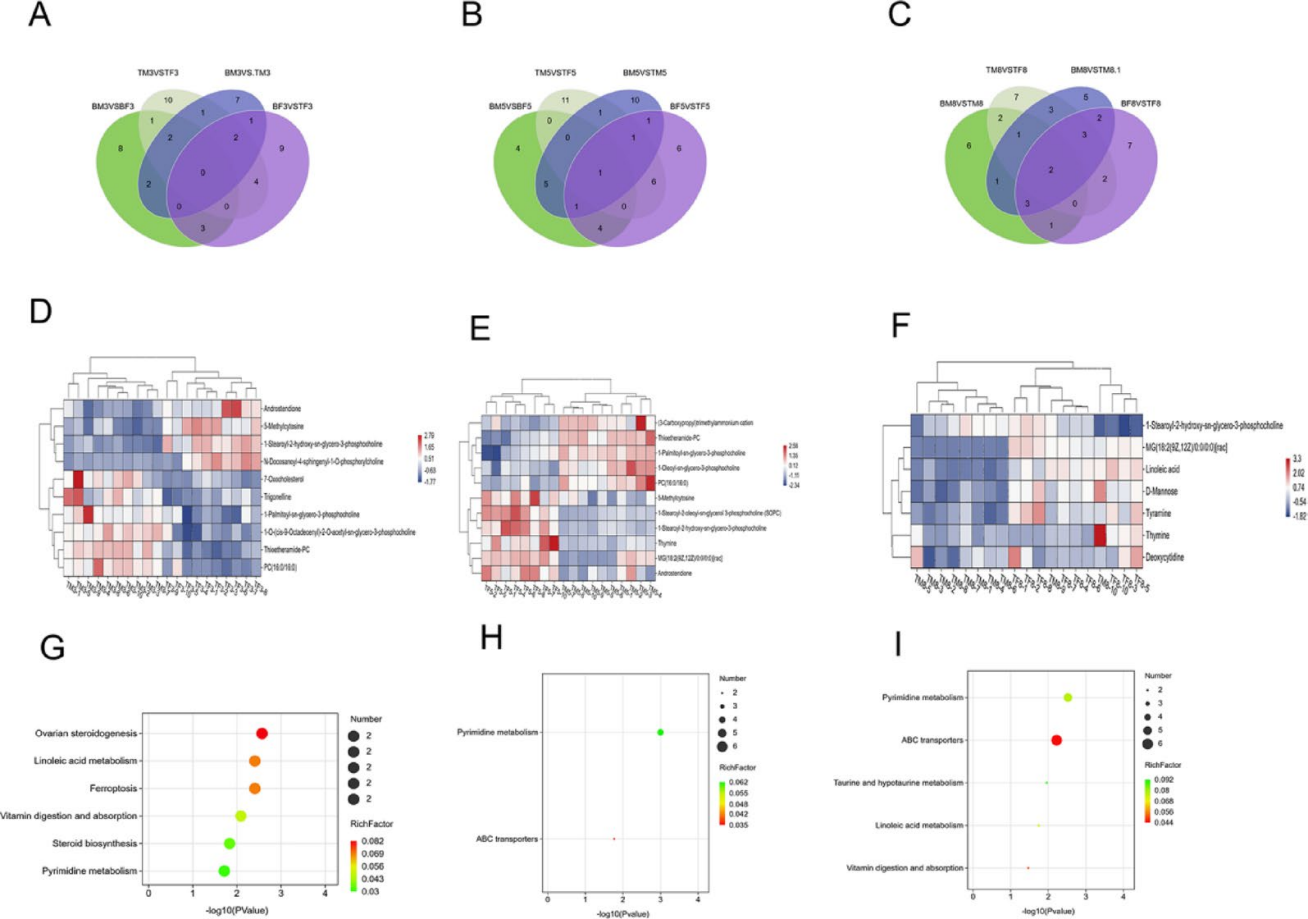
Metabolomics of male and female rats in the experimental group at 30 min, 2 h, and 12 h Control group male and female rats, experimental group male and female rats, male rats in both experimental and control groups, and female rats in both experimental and control groups. Metabolites differing between groups were identified using Venn diagrams to determine intersections. Green represents male and female rats in the control group, white represents male and female rats in the experimental group, blue represents male rats in both experimental and control groups, and purple represents female rats in both experimental and control groups. **A**: 30 min, **B**: 2 h, **C**: 12 h. Heatmap of differentially metabolized compounds in male and female experimental rats, Blue indicates reduced expression levels, white indicates no regulation, and red indicates increased expression levels. **D**: 30 min, **E**: 2 h, **F**: 12 h. Perform metabolic pathway analysis on the filtered differentially expressed metabolites using the KEGG database. **G**: 30 min, **H**: 2 h, **I**: 12 h

We performed pairwise comparisons of metabolites between male and female rats in the experimental and control groups. After excluding the differential metabolites between male and female rats in the control group and the differential metabolites between male and female rats before and after administration (Fig. 6A-F, specific compounds provided in Appendix 5). We performed KEGG enrichment analysis on metabolites that differed between male and female rats in the experimental group (Fig. 6G-I). The results showed that 6 pathways were mainly enriched at 30 min, 2 pathways were enriched at 2 h, and 5 pathways were enriched at 12 h (Table 4).

**Table 4:** KEGG pathway of experimental male and female rats

Time	Pathway name	Test	PValue	Richfactor
30 min	Ovarian steroidogenesis	2	0.006	0.083
	Linoleic acid metabolism	2	0.008	0.071
	Ferroptosis	2	0.008	0.069
	Vitamin digestion and absorption	2	0.015	0.051
	Steroid biosynthesis	2	0.031	0.034
	Pyrimidine metabolism	2	0.038	0.031
2 h	Pyrimidine metabolism	4	0.001	0.062
	ABC transporters	2	0.017	0.035
12 h	Pyrimidine metabolism	5	0.003	0.077
	ABC transporters	6	0.006	0.044
	Taurine and hypotaurine metabolism	2	0.011	0.091
	Linoleic acid metabolism	2	0.018	0.071
	Vitamin digestion and absorption	2	0.034	0.051

## Discussion

In this study, we established a rat intoxication model to determine sex-specific differences in PKs and metabolomics associated with acute strychnine intoxication. Strychnine metabolism rates differed between male and female rats despite identical dosing. In intact rats, the peak concentration of drug in female rat was higher than that in male rat. After gonadectomy, the peak concentration of male and female rats was significantly lower than that in the intact group, indicating that sex hormones have an effect on the drug metabolism of strychnine. Yu investigated the gender differences in the pharmacokinetics and metabolism of VX-548 in rats. After administration, the AUC of VX-548 in female rats (1505.8 ± 47.3 ng·h/mL) was significantly higher than that in male rats (253.8 ± 6.3 ng·h/mL), and the clearance rate in female rats (12.5 ± 0.8 mL/min/kg) was significantly lower than that in male rats (65.1 ± 1.7 mL/min/kg) [16]. Zhang have previously reported the differential effects of exogenous testosterone administration on the toxicokinetics and toxicodynamics of γ-aminobutyric acid. Following gonadectomy, testosterone-treated male rats exhibited significantly lower renal clearance and higher area under the curve than female rats with similar treatment [17]. Jin explored the changes in plasma digoxin concentration in ovariectomized rat and found that the peak digoxin concentration was significantly lower after ovariectomy [18]. This change was attributed to decreased estrogen levels, which matched our experimental results. Female rats have a higher fat content, and fat tissue can serve as an additional storage reservoir, resulting in more complex drug metabolism. In this experiment, the pharmacokinetics of intact male rats were best described by a two-compartment model, while intact female rats and GDX female rats both followed three-compartment kinetics. In contrast, GDX male rats exhibited one-compartment model kinetics. These compartmental distinctions suggest significant sex-dependent differences in strychnine metabolism. Rivera found that female gender is associated with lower intercompartmental clearance, which may explain why females require a three-compartment model (e.g. due to deeper tissue compartments), while males, with higher clearance rates, may be simplified to a one or two-compartment model [19]. Therefore, females may require a three-compartment model due to hormonal, distribution volume, and clearance rate differences, while males, with more efficient metabolism and clearance, may be simplified to a one- or two-compartment model.

Further, we found that the peak plasma concentration of strychnine in GDX female rats was 3.70 times that of GDX male rats, indicating the influence of factors other than sex hormones in strychnine metabolism. Next, we studied strychnine metabolomics in intact male and female rats. A total of 19 metabolites potentially associated with differential strychnine metabolism in male and female rats were identified through careful screening. The metabolite types primarily included cholesterol and inorganic acids, followed by pyrimidines and pyrimidine analogs. The types of differential metabolites varied considerably with time after drug administration. KEGG metabolic pathway analysis identified 9 metabolic pathways potentially associated with the sex-specific differences in strychnine metabolism. Notably, the metabolites involved with these pathways also varied at different time points after drug administration.

ABC transporters are among the oldest known transporter protein families and play an important role in transmembrane substance transport. They can transport inorganic and organic small molecules such as sugars, metal ions, and amino acids, as well as organic macromolecular compounds such as proteins, oligonucleotides, and cellular metabolites [20]. The differential expression of ABC transporters in this study was reflected in the up-regulation of various metabolites, such as deoxycytidine and D-mannose at 12 h after poisoning with strychnine. In fact, the overall metabolic pathway was up-regulated, suggesting that the expression of ABC transporters was relatively stronger in female rat than that in male rat. ABC transporters proteins maintain stability in the intracellular environment by transporting various substances across the cell membrane, crucial for drug absorption and distribution [21–23]. Their expression and activity are regulated by a variety of signaling pathways, particularly the estrogen receptor signaling pathway, which plays an important role in the transport, catabolism, and mRNA expression of transporter proteins [24]. Differential activation of this pathway in male and female rats may be responsible for the differential expression of ABC transporters, explaining the differences in strychnine absorption and distribution rates between sexes.

Linoleic acid can inhibit the accumulation of cholesterol in body, maintaining stable levels [25–27]. Alteration in linoleic acid metabolism affects overall lipid metabolism and cholesterol homeostasis in the body. Through steroid biosynthesis, cholesterol is converted to pregnenolone and then to various hormones, which is important to maintain normal physiological state of body [28–30]. Bile acids are steroidal carboxylic acids derived from vertebrate cholesterol. Primary bile acids, such as cholic and goose deoxycholic acids, are synthesized in the liver, combined with taurine or glycine, then secreted into the intestinal tract through bile. Bile acids are metabolites of cholesterol in liver cells and play a crucial role in fat metabolism. In this study, relevant bile acid metabolites were higher in female rat than that in male rat, suggesting that strychnine affects fat metabolism by interfering with bile acid metabolism. The level of 1-Stearoyl-2-hydroxy-sn-glycero-3- phosphocholine and Linoleic acid in plasma increased in female rat than those in male rat at 30 min and 12 h of strychnine administration, suggesting that the linoleic acid metabolic pathway was activated at these time points. Linoleic acid content is higher in female rat than that in male rat. Its integration with cell membrane phospholipids increases membrane fluidity, accelerates transmembrane drug transport, shortens the time to peak, and increases peak concentration. Furthermore, it delays drug release from the membrane, prolonging its duration of action [31, 32]. These phenomena may be the reason for the difference in peak concentration and metabolism rate of strychnine in male and female rat. In female rat, strychnine disrupted linoleic acid metabolism to a greater extent than that in male rat, indicated a higher effect on female rat than that on male rat.

Pyrimidine is an essential substrate involved in nucleic acid, phospholipid, and glucose metabolism, as well as in protein glycosylation. Pyrimidine metabolism plays a decisive role in biological processes such as cell proliferation, differentiation, apoptosis, and metastasis [33, 34]. Disorders of pyrimidine metabolism restrict DNA, RNA, and protein synthesis, affecting the normal function of cells and resulting in cell death [35]. This effect is clinically utilized in cancer treatment to inhibit cancer cells and could be the primary mechanism underlying the anti-tumor and anti-cancer function of strychnine [5, 36, 37]. In present study, pyrimidine metabolism was observed at 30 min, 2 h, and 12 h post-strychnine treatment in rat, indicating that pyrimidine metabolism occurred throughout the metabolic process of strychnine. Additionally, metabolites such as 5-methylcytosine, and thymine were up-regulated, suggesting that the degree of disturbance of pyrimidine metabolism by strychnine in females might be higher than that in males. In other words, strychnine has higher toxicity in females than in males. Furthermore, 5-methylcytosine and other metabolites produced by pyrimidine metabolism can activate the cellular stress pathway and up-regulate the expression of ABC transporter proteins. The synergistic effect can influence the uptake and distribution rates of strychnine in male and female rats.

This study has several limitations. First, it focused solely on investigating the metabolic differences of strychnine between male and female rats, missing investigation of potential species differences and lacking human data. Future research should incorporate blood samples from human subjects of different genders exposed to strychnine to analyze gender-specific metabolic variations. The secretion of sex hormones is also affected by age. Future experimental design should take into account the effect of age on the absorption and metabolism of sex hormones. Such investigation would enhance the metabolic dataset and provide valuable insights for personalized clinical medication strategies. Second, the investigation employed only pharmacokinetics and untargeted metabolomics approaches, lacking specificity. Future studies could utilize multi-omics technologies to further elucidate the molecular mechanism underlying the gender-specific effects of strychnine.

## Conclusion

Altogether, the PKs and metabolomics data highlight the significant differences between male and female rats. These differences are reflected in key metabolic pathways, such as amino acid and lipid metabolism, and are closely related to health, playing an important role in tumors and related metabolic and immune responses.

In summary, sex-dependent differences in strychnine metabolism were observed in rat models of acute exposure. Gonadectomy substantially altered the metabolic profile, indicating potential regulation by sex hormones. These findings underscore the necessity for sex-specific dosing strategies to optimize therapeutic efficacy and patient prognosis. Given the higher systemic exposure and reduced clearance in females, we propose that dosage adjustment (e.g., reduction for female patients) may mitigate accumulation-related toxicity during clinical management of strychnine poisoning.

## Supplementary Information

Supplementary Material 1.

Supplementary Material 2.

Supplementary Material 3.

Supplementary Material 4.

Supplementary Material 5.

## Data Availability

Data is provided within the manuscript or supplementary information files.
